# Dr. Breima

**Published:** 1890-08

**Authors:** 


Dr. Breima recommends the use of the following in chronic gonorrhea as an injection:

| | |
|---|---|
| ℞—Creosote | *m* x. |
| Ext. fl. hamamelis, | |
| Ext. fl. hydrast. canaden. | aa *m* xv |
| Aquæ rosæ | ℥ xxxij. |

M. Sig.—Dilute with warm water before using.

